# Targeting IKKβ in Cancer: Challenges and Opportunities for the Therapeutic Utilisation of IKKβ Inhibitors

**DOI:** 10.3390/cells7090115

**Published:** 2018-08-23

**Authors:** Jack A. Prescott, Simon J. Cook

**Affiliations:** Signalling Laboratory, The Babraham Institute, Babraham Research Campus, Cambridge CB22 3AT, UK

**Keywords:** IKK, inhibitory kappa B kinase, nuclear factor kappa B, inflammation, cancer, small molecule kinase inhibitors, therapeutics

## Abstract

Deregulated NF-κB signalling is implicated in the pathogenesis of numerous human inflammatory disorders and malignancies. Consequently, the NF-κB pathway has attracted attention as an attractive therapeutic target for drug discovery. As the primary, druggable mediator of canonical NF-κB signalling the IKKβ protein kinase has been the historical focus of drug development pipelines. Thousands of compounds with activity against IKKβ have been characterised, with many demonstrating promising efficacy in pre-clinical models of cancer and inflammatory disease. However, severe on-target toxicities and other safety concerns associated with systemic IKKβ inhibition have thus far prevented the clinical approval of any IKKβ inhibitors. This review will discuss the potential reasons for the lack of clinical success of IKKβ inhibitors to date, the challenges associated with their therapeutic use, realistic opportunities for their future utilisation, and the alternative strategies to inhibit NF-κB signalling that may overcome some of the limitations associated with IKKβ inhibition.

## 1. Introduction

There are five members of the nuclear factor ‘kappa-light-chain-enhancer’ of activated B-cells (NF-κB) transcription factor family in mammals: RelA (p65), RelB, c-Rel, NF-κB1 (p50, initially synthesised as a larger precursor, p105) and NF-κB2 (p52, initially synthesised as a larger precursor, p100). All members share a conserved Rel homology domain (RHD) that enables them to associate with each other to form a diverse array of transcriptionally active homo- and hetero-dimeric complexes. In normal unstimulated cells, NF-κB dimers are tightly bound by members of the inhibitor of kappa B (IκB) family of proteins, which maintain the cytoplasmic steady-state localisation of NF-κB dimers and inhibit their DNA-binding activity. The precursor proteins p100 and p105 also contain ankyrin repeat domains, which are cleaved upon processing to p52/p50, such that they comprise internal inhibitors of NF-κB dimers. 

**Figure 1:**
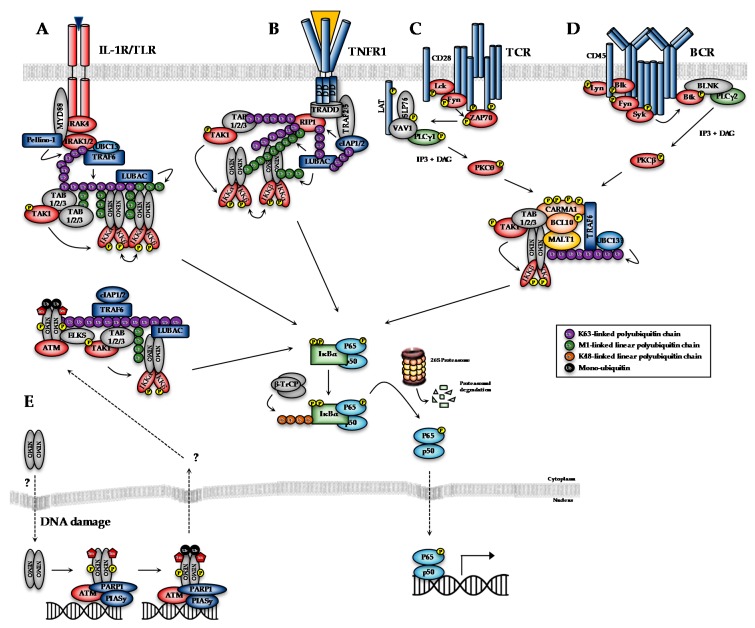
Overview of canonical and DNA damage-induced NF-κB signalling pathways. (**A**) Binding of IL-1/Toll-like receptor (TLR) ligands to the interleukin-1 receptor (IL-1R)/TLRs leads to the assembly of the so-called ‘Myddosome’, an oligomeric structure consisting of the adaptor protein MyD88, IL-1 Receptor (IL-1R)-Associated Kinase 4 (IRAK4), IRAK1 and IRAK2. IRAK4 activates IRAK1, allowing IRAK1 to autophosphorylate and subsequently phosphorylate the E3-ligase Pellino-1, which in turn causes K63-polyubiquitylation of IRAK1. This leads to the recruitment and activation of TRAF6, which along with the E2-conjugating complex Ubc13-Uev1a, generates K63-linked polyubiquitin chains that serve to recruit and activate the TAK1 complex or TAB1/2/3-TAK1. K63-linked chains also serve as a substrate for the LUBAC (linear ubiquitin assembly complex) complex, which conjugates M1-linked ubiquitin to these oligomers, to generate M1-K63-linked hybrid ubiquitin chains. The IKK complex is recruited to this complex through interaction of NEMO with M1-linked chains. The co-localisation of TAK1 and IKK to ubiquitin chains leads to activation of the IKK complex, which subsequently phosphorylates IκBα to activate the NF-κB pathway. (**B**) TNFα binding to the extracellular domain of the receptor leads to the recruitment of TRADD (Tumor necrosis factor receptor type 1-associated DEATH domain) to the cytoplasmic death domains of TNFR1. TRADD, in turn, recruits RIP kinase, and subsequently TRAF2 or TRAF5 adaptor proteins and cIAP1 or cIAP2 to assemble the TNFR1 complex I. cIAP1 and cIAP2 generate K63-linked polubiquitin chains on RIP1 and other components of the complex. This is necessary to recruit LUBAC, which stabilises complex I by catalysing the attachment of linear M1-linked polyubiquitin chains, typically to RIP1. K63-polyubiquitylated RIP1 also recruits the TAK1:TAB complex. LUBAC-mediated M1-linked linear polyubiquitylation of RIP1, meanwhile promotes the recruitment of NEMO, as part of the IKK complex. Membrane proximal recruitment of IKK kinases contributes to IKK activation through proximity to TAK1, which is thought to prime the activation of IKK via phosphorylation of S176/S177 of IKKα/IKKβ, and through oligomerisation of IKK complexes, which is thought to facilitate trans-autophosphorylation of the activation loop, leading to full activation. (**C**) Engagement of the TCR by a major histocompatibility complex (MHC)-antigen complex leads to recruitment of Src family kinases, including FYN and LCK, which phosphorylate the TCR to promote recruitment of the tyrosine kinase, ZAP-70. ZAP-70 phosphorylates the adapter proteins LAT and SLP-76, which along with VAV1 promote the recruitment and activation of PLCγ1. PLCγ1 generates the second messengers, inositol trisphosphate (IP3) and diacylglycerol (DAG), which, in turn, activate a specific PKC isoform, PKCθ. PKCθ-mediated phosphorylation of CARMA1 triggers a conformational change, enabling CARMA1 to bind to BCL10 and MALT1, to form the CBM complex. BCL10 and MALT1 become polyubiquitinated, possibly through TRAF6 activity, which promotes the recruitment of NEMO, as part of the IKK complex. (**D**) Antigen binding to BCRs leads to the recruitment and activation of SRC-family kinases, including BLK, LYN, FYN and SYK and adaptors, such as BLNK. This leads to activation of PLCγ2, which catalyses the generation of IP3 and DAG, which ultimately activate a specific PKC isoform, PKCβ. PKCβ phosphorylates CARMA1 to form the CBM complex and ultimately activate the IKK complex. **(E)** Genotoxic triggers the nuclear accumulation of ‘IKK-free’ NEMO. Within the nucleus NEMO forms a complex with PARP1, PIASy and ATM and undergoes a series of post-translational modification. PIASy promotes the sumoylation of NEMO, which promotes its nuclear localisation. ATM phosphorylates NEMO at Serine 85, which is necessary for the subsequent monoubiquitylation of NEMO. This is thought to trigger the nuclear export of the NEMO-ATM complex, which then, in an ill-defined mechanism, activates TAK1 and the IKK complex. Canonical and DNA damage-induced NF-κB signalling pathways converge at the activation of the IKK complex, which subsequently phosphorylates IκB proteins (at S32 and S36 IκBα). This promotes the recognition of the PEST motif degron within IκBα by β-TrCP, which is part of the E3 ubiquitin ligase SCF^β-TrCP^ (S phase kinase-associated protein 1 (SKP1)-cullin 1-F-box protein containing β-transducing repeat-containing protein), and its K48-linked ubiquitylation, which targets IκBα for proteasomal degradation. This enables NF-κB complexes (primarily p65-p50 and c-rel/p50 complexes in the case of canonical NF-κB pathways), to accumulate in the nucleus, where they regulate the expression of NF-κB-dependent genes.

The transcriptional activity of NF-κB dimers is regulated by several distinct pathways (Figure 1). The first is the canonical pathway, which is induced by pro-inflammatory cytokines, such as tumour necrosis factor-alpha (TNF-α) and interleukin-1 (IL-1), engagement of antigen receptors, such as the T- and B-cell receptor (T/B-CR), pathogen-associated molecules such as lipopolysaccharides (LPS) and certain growth factors (Figure 1A–D; [1]). Engagement of the canonical NF-κB pathways triggers signalling cascades that converge on activation of the IκB kinase (IKK) complex, which is formed by the kinase subunits, IKKα and IKKβ, and a regulatory subunit, IKKγ (also known as NEMO, NF-κB essential modifier). The non-canonical pathway, meanwhile, is stimulated by a more restricted set of cytokines all belonging to the TNF superfamily, including BAFF (B-cell activating factor) and lymphotoxin β [2]. Non-canonical NF-κB signalling requires IKKα, but not NEMO or IKKβ activity, and so will not be discussed in detail here. Several atypical pathways are also capable of activating the IKK complex in response to diverse stresses, such as DNA damage (Figure 1E; [3]). These distinct NF-κB pathways regulate different subsets of target genes, and hence different biological functions. 

**Figure 2:**
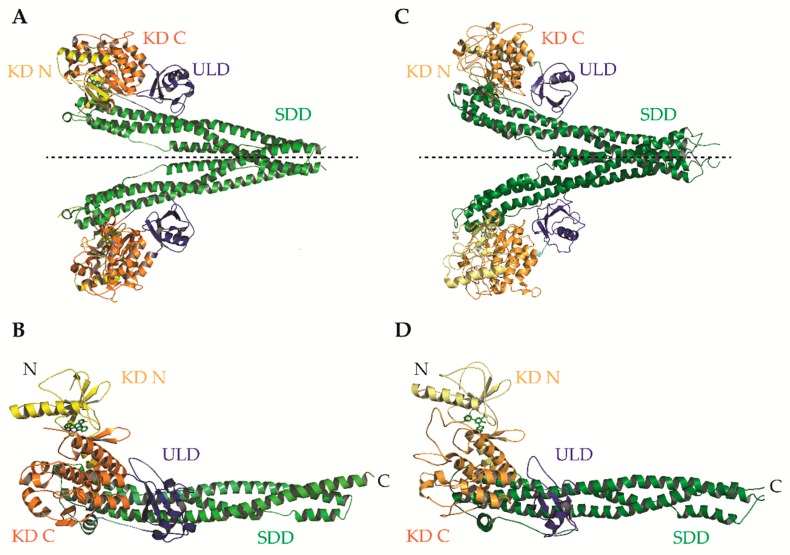
X-ray crystal structures of human IKKβ and IKKα. (**A**) Ribbon diagram of the crystallographic structure of the human IKKβ dimer. The N-terminal lobe of the kinase domain (KD) (KD N; residues 1–109), C-terminal lobe of the KD (C; residues 110–307), ubiquitin-like domain (ULD; residues 308–404) and scaffold/dimerization domain (SDD; residues 410-664) are coloured in yellow, orange, blue and green, respectively. The NEMO-binding domain (NBD) at the extreme N-terminus was not resolved in the original structure. Figure adapted from [5]. PDB ID: 4KIK. (**B**) Ribbon diagram of the human IKKβ protomer showing the tri-modular architecture of KD, ULD, and the elongated, α-helical SDD. Figure adapted from [5]. PDB ID: 4KIK. (**C**) Ribbon diagram of a model of a human IKKα dimer derived from X-ray crystallographic data. Figure adapted from [7]. PDB ID: 5EBZ. (**D**) Ribbon diagram of the human IKKα protomer showing the same tri-modular architecture of domains as IKKβ. The NBD at the extreme N-terminus was also not resolved in the original structure. Figure adapted from [7]. PDB ID: 5EBZ. The pseudo two-fold axis of the IKKβ and IKKα dimers are indicated by a dashed line.

IKKα and IKKβ are ubiquitously expressed serine/threonine kinases with 52% sequence identity and 70% homology [4]. They also share highly similar domain organisation and tertiary structure, as demonstrated by the recent X-ray crystal structures of human IKKα and IKKβ (Figure 2; [5,6,7]. Activation of IKKα and IKKβ kinase activity requires the phosphorylation of specific residues in the activation loop of their active sites: serine-176 (S176) and serine-180 (S180) for IKKα and S177, and S181 for IKKβ [8,9]. The precise sequence of molecular events involved in IKK activation remain to be fully determined. However, recent evidence for higher-order IKK complexes [10], combined with X-ray crystal structures of IKKβ dimers in catalytically active conformations [6], has led to the proposal of a model for IKK activation involving oligomerization-mediated trans-autophosphorylation of IKK subunits. Indeed, in the case of IKKβ activation downstream of IL-1 and TNFα in mouse embryonic fibroblasts (MEFs) and TLR ligands in macrophages, TAK1 has been proposed to phosphorylate IKKβ at S177, which primes subsequent IKKβ-catalysed autophosphorylation of S181 [11]. Subsequently, the activated IKK complex phosphorylates IκB proteins (at S32 and S36 in the case of IκBα), promoting their K48-linked ubiquitylation by the S phase kinase-associated protein 1 (SKP1)-cullin 1-F-box protein (SCF)/beta-transducing repeat-containing protein (β-TrCP) E3 ubiquitin ligase complex, which targets them for proteasomal degradation [12,13,14,15,16]. In the context of canonical NF-κB signalling, this enables RelA- and c-Rel-containing NF-κB dimers to accumulate in the nucleus where they coordinate the expression of genes involved in diverse biological process including cell proliferation/survival, immune and inflammatory responses, and other host defence mechanisms [17].

The activity of NF-κB subunits is also regulated through direct post-translational modification including phosphorylation, ubiquitination, sumoylation, acetylation and nitrosylation [18,19]. In many cases, these modifications are mediated by IKKα and IKKβ, as well as components of heterologous signalling pathways. This provides an additional layer of fine control through which NF-κB transcriptional activity can be modulated and represents a key site of cross-talk within the wider signalling network [3].

NF-κB signalling pathways are normally tightly controlled by multiple regulatory mechanisms to ensure minimal basal activation [20]. However, given its critical role in regulating the expression of genes involved in cell-survival, proliferation, angiogenesis, metabolism, inflammation and cell adhesion/migration, it is unsurprising that a wide range of inflammatory diseases and cancers have been shown to exhibit deregulated NF-κB signalling that results in constitutive pathway activation [21]. Indeed, the ability of NF-κB to induce inflammation places it as one of the crucial links between chronic inflammation and cancer [22]. However, aberrant NF-κB activation is also capable of promoting tumorigenesis in cancers whose early progression isn’t typically associated with inflammation through contributions of NF-κB target genes to almost all the hallmarks of cancer [23,24]. 

Outside of their direct role in the NF-κB signalling pathway, both IKKα and IKKβ have been proposed to phosphorylate an ever-growing list of ‘non-classical’ substrates involved in diverse biological processes [25]. Whilst, many of these ‘non-classical’ phosphorylation events and the associated NF-κB-independent functions of IKKs await thorough validation, it is clear that the IKK complex is a central point of cross-talk between NF-κB and other signalling pathways. Many of the substrates phosphorylated by IKKα and IKKβ, such as FOXO3a and TSC1, are involved in proliferative and pro-survival pathways, and so these NF-κB-independent functions of the IKKs may also contribute to tumorigenesis [26,27].

## 2. IKKβ Inhibitors

The identification of NF-κB signalling pathways as important drivers of human disease and the essential role of the IKK complex as the ubiquitous signal integration hub for NF-κB activation pathways led to a concerted effort by the pharmaceutical industry to identify small molecule inhibitors of the IKKs via high-throughput screening programmes. The primary focus of efforts to inhibit canonical NF-κB signalling has been the druggable IKKβ kinase due to foundational mouse studies that indicated IKKβ and NEMO are essential for activation of this pathway, while IKKα is largely dispensable [28,29,30]. Instead, IKKα, but not IKKβ or NEMO, is indispensable for non-canonical NF-κB activation [31,32,33,34].

A vast drug-discovery effort has identified numerous synthetic small molecules with activity against the IKKs that typically exhibit selectivity for IKKβ over IKKα. Indeed, the first IKKα-selective inhibitor series was only very recently reported [35] and IKKα-selective inhibitors are described in another review within this special issue by Pepper & Mackay. In addition, a range of natural products, such as wedelactone, reportedly inhibit IKKβ [36,37]. However, these compounds tend to have pleiotropic effects, and with a few notable exceptions, their mechanism of IKK/NF-κB inhibition has not been fully characterised. Collectively, the number of compounds with reported activity against IKKβ continues to grow [38,39]. A selection of the best-characterised, commercially-available IKKβ inhibitors is shown in Table 1. 

To date, chemical IKKβ inhibitors with four mechanisms of action have been characterised (see Table 1). The clear majority are ATP analogues that exhibit reversible, ATP-competitive activity, with some degree of selectivity for IKKβ over IKKα and other kinases. Due to the structural similarity of protein kinase ATP-binding sites, ATP mimetics often exhibit activity against other kinases, resulting in ‘off-target’ effects at or near concentrations of drug required to inhibit its primary target in cells [96]. Indeed, several widely-used ‘selective’ IKKβ inhibitors have recently been shown to exhibit significant off-target effects. For example, the compound Bay 11-7082, which inhibits IκBα phosphorylation and NF-κB transcriptional activity in cells, has been used to study IKK and NF-κB function in >350 publications [97]. However, a recent report showed that Bay 11-7082 inhibits NF-κB not through direct inhibition of IKK activity, but through irreversible covalent inactivation of the E2-conjugating enzymes Ubc (ubiquitin conjugating) 13 and UbcH7, and the E3-ligase LUBAC (linear ubiquitin assembly complex) [98]. In addition, the widely used compound TPCA-1 was recently shown to inhibit STAT3 signalling through direct binding to the STAT3 Src Homology 2 (SH2) domain, in addition to its activity against IKKβ [51]. Of great concern is the continued use of these non-selective inhibitors to make inferences about IKK function. The current best-in-class, ATP-competitive IKKβ inhibitors are MLN-120B and BI605906, which exhibit >50- and >300-fold selectivity for IKKβ over IKKα, respectively [40,41,99]. The selectivity of BI605906 for IKKβ over 100 other serine/threonine and tyrosine kinases has also been confirmed [40], while MLN-120B exhibited a highly favourable selectivity profile when tested against a panel of 442 kinases [99], making these inhibitors the preferred tools for dissecting IKKβ-dependent functions [100]. 

Meanwhile, BMS-345541 acts as an allosteric inhibitor of the IKKs, displaying moderate (13-fold) selectivity for IKKβ over IKKα [83]. Curiously, BMS-345541 binds to IKKβ in a mutually exclusive manner with respect to phosphorylated IκBα substrate and in a non-mutually exclusive manner with respect to ADP. Binding to IKKα has the opposite effect, leading to a proposed binding model whereby BMS-345541 binds to similar allosteric sites on IKKα and IKKβ but affects the active sites of the subunits differently. 

A third class of IKKβ inhibitors constitute thiol-reactive compounds that interact with key cysteine residues in IKKβ. For example, berberine [101], nimbolide [102] and withaferin A [92] have been proposed to inhibit IKKβ activity through covalent modification of cysteine 179 (C179), a residue that may promote phosphorylation of ser-177/Ser-181 and, in turn, kinase activity [103]. However, the involvement of C179 modification in the mechanism of action of withaferin A has been questioned recently by a study that found it to be a poor direct inhibitor of IKKβ in an in vitro kinase assay; instead it was proposed to inhibit IKKβ indirectly through blockade of signal-induced NEMO reorganization into ubiquitin-based signalling foci [89]. Other molecules, such as the epoxyquinoid derivatives, manumycin A [104] and jesterone dimer [105], are thought to inhibit IKKβ activity through covalent cross-linking of IKKβ monomers via C179, which disrupts the essential interaction between IKKβ and NEMO. Modification of C179 may also block S-glutathionylation at this residue, which is thought to be important for the kinase activity of IKKβ [106]. The natural product ainsliadimer A, meanwhile, covalently modifies the conserved residue, cysteine-46, in both IKKα and IKKβ, to inhibit ATP-binding and kinase activity via a putative allosteric mechanism [95]. A fourth mechanism of action for IKKβ inhibitors has been proposed for the benzoxathiole derivate, BOT-64, which inhibited IKKβ kinase activity via an apparent direct interaction with S177 and/or S181 residues in the activation loop of IKKβ [94], although 10 years on from the primary paper further work is required to validate this. 

In addition, IKKβ kinase activity may be inhibited indirectly through blockade of the essential interaction between IKKβ and NEMO. For example, cell-permeable peptides corresponding to the NEMO-binding domain (NBD) of IKKα/β display non-selective inhibitory activity against IKKα and IKKβ [107]. These molecules will be discussed further in Section 7.2.

Multiple clinically approved non-steroidal anti-inflammatory agents (NSAIDs), including sodium salicylate (aspirin), sulindac sulphide and exisulind, have also been proposed to inhibit the NF-κB pathway at the level of IKKβ. The primary anti-inflammatory mechanism of these compounds is via inhibition of the cyclooxygenase enzymes, COX1 and COX2 [108]. However, these compounds have also been reported to inhibit NF-κB activation by inhibiting IκBα phosphorylation [109,110,111,112], while at higher concentrations aspirin has been proposed to act as an ATP-competitive inhibitor of IKK-β (in vitro IC_50_ ~ 80µM) [110,112]. However, these findings should be interpreted with extreme caution as more recent kinase profiling studies suggest that aspirin inhibits vast numbers of other kinases at least as potently as it inhibits IKKβ [113]. Furthermore, some studies have suggested that the direct inhibition of IKKβ activity by aspirin in vitro does not reflect its inhibitory mechanism in vivo, where aspirin is proposed to inhibit TNFα-induced IκBα phosphorylation and degradation indirectly through activation of p38 kinase [114,115]. In addition, more recent studies have proposed that while short-term treatment (1–2 h) with NSAIDs may block stimuli-induced NF-κB activation in cells, prolonged exposure with pharmacologically relevant doses of NSAIDs, in fact, stimulates the NF-κB pathway both in vitro and in vivo ([116,117,118,119,120]. Furthermore, this activation has been causally associated with pro-apoptotic effects of these agents in cancer cells and may involve NSAID-induced nucleolar translocation of RelA [121].

## 3. Pre-clinical Development of IKKβ Inhibitors

IKKβ inhibitors have demonstrated efficacy in various pre-clinical models of cancer and inflammatory disease (see Table 1). For instance, MLN-120B inhibited multiple myeloma (MM) cell growth in a clinically relevant severe combined immunodeficient (SCID)-hu mouse model [42] and exhibited significant therapeutic efficacy in a rat model of rheumatoid arthritis (RA) [43]. However, clinical use of these inhibitors has not yet been reported. Indeed, only a handful of phase I/II clinical trials with IKK inhibitors have been performed. The earliest example was the ATP-competitive IKKβ inhibitor, MLN-0415, which failed in phase I human trials for inflammatory disorders due to an unfavourable safety profile [122]. It is now being tested in dogs with high-grade lymphomas. The *Institute of Medicinal Molecular Design Inc* (IMMD) has several compounds in current or completed phase I/II trials, for which results have yet to be published [122]. For example, IMD-2560 (a pro-drug of IMD-0560) [123] underwent a phase I trial for the treatment of rheumatoid arthritis (RA), rheumatic osteoporosis and osteoarthritis, IMD-0354 [124] underwent a phase I trial for the topical treatment of atopic dermatitis and IMD-0354 (and its pro-drug IMD-1041) underwent a proof-of-concept (POC) study for the treatment of chronic obstructive pulmonary disease (COPD; Identifier: NCT00883584). However, questions surrounding the true molecular target of these compounds has been raised by the lack of conclusive biochemical evidence for IKKβ antagonism. Indeed, IMD-0354 was recently shown to exhibit no activity against IKKβ or IKKα in an ATP-based kinase assay [125].

The IKKβ-selective compound, SAR-113945 [126], has progressed the furthest through clinical development. Multiple phase I trials demonstrated its safety/tolerability following intra-articular injection in patients with knee osteoarthritis (Identifier: NCT01113333/NCT01463488/ NCT01511549). Positive efficacy trends in these studies motivated the undertaking of a Phase IIa POC trial (Identifier: NCT01598415). However, SAR-113945 failed to show efficacy in this larger patient sample size [127].

The ATP-competitive IKKβ inhibitor, SPC-839 (also known as AS602868; [128,129]), also progressed into phase I trials for haematological malignancies, but the trial was prematurely terminated due to portfolio repositioning. This reflects a common trend within the pharmaceutical industry; interest in the clinical development of IKKβ-selective inhibitors has significantly diminished in the last 10 years.

## 4. Potential Reasons for the Lack of Clinical Success of IKKβ Inhibitors

**Figure 3:**
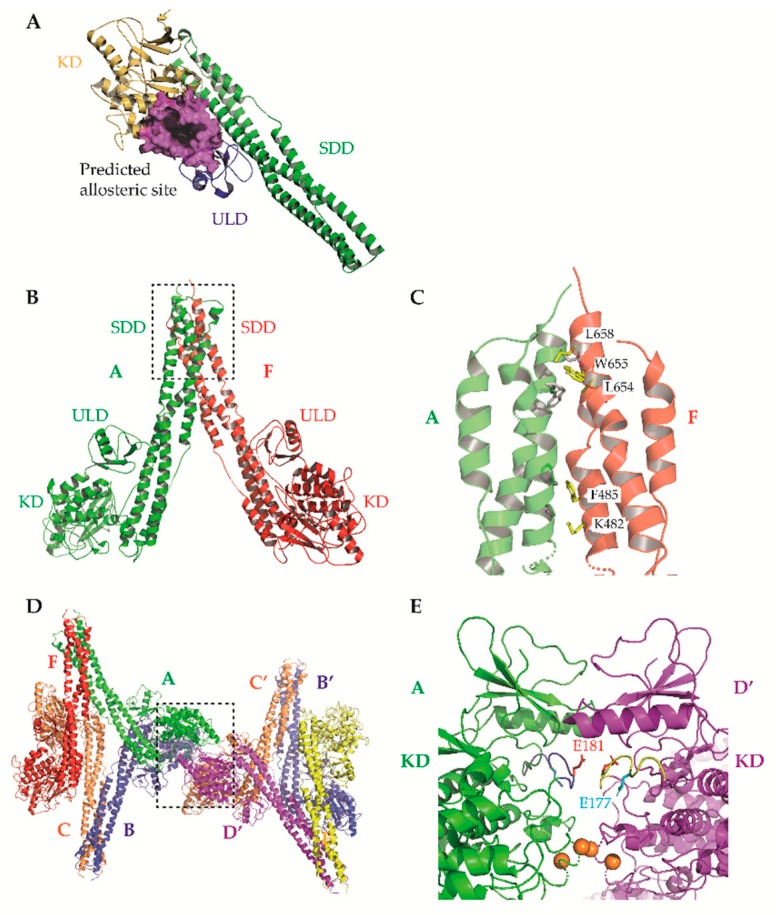
Insights from X-ray crystallographic studies of IKKβ. (**A**) Predicted allosteric binding site between the KD (yellow) and ULD (blue) of the catalytically inactive conformation of the human IKKβ monomer. Surface representation of residues surrounding the binding pocket is shown in magenta. SDD, green. Liu et al. identified a compound that specifically binds to this allosteric pocket in the inactive conformation of IKKβ, but not the active conformation, and blocks IKKβ activation. Figure adapted from [130] PDB ID: 4KIK. (**B**) Ribbon diagram of a human IKKβ dimer (chains A and F) in a catalytically active conformation taken from the asymmetric unit of the crystallographic structure. The primary dimer interface is mediated by residues of the C-terminal portion of the SDD (dashed box). Figure adapted from [6]. PDB ID: 4E3C. (**C**) Close-up view of the boxed area from panel B. Displayed are residues mediating interactions at the dimer SDD interface that have been shown to be important for IKKβ catalytic activity in vitro via site-directed mutagenesis. Three pairs of residues were mutated (W655D/L658D, L654D/W655D and K482A/F485D) and in vitro kinase assays with human IKKβ performed [6]. Figure adapted from [6]. PDB ID: 4E3C. (**D**) Ribbon diagram showing the interaction of neighbouring, symmetry-related tetrameric assemblies of IKKβ protomers within the crystal. This oligomerisation positions two KDs (from chains A, green, and D’, magenta, in the representation provided) within close proximity to one another (dashed box). Figure adapted from [6]. PDB ID: 4E3C. (**E**) Close-up view of the boxed area from panel D. The arrangement of neighbouring KDs (from A and D’) positions the kinase activation loop (shown in yellow and blue) of one protomer directly over the active site of its neighbour, and potentially facilitates oligomerization-dependent trans auto-phosphorylation. Activation loop E177 and E181 (mutant forms of WT S177 and S181) are shown in cyan and red, respectively. The Cα positions of V229 and H232 are marked as orange spheres. Mutation of these, and other residues mediating interactions at this KD-KD oligomerisation interface inhibited IKKβ catalytic activity and activation loop phosphorylation in vitro [6]. Figure adapted from [6]. PDB ID: 4E3C. Small molecules designed to interfere with dimerization/oligomerization via the interfaces shown in panel C and E may function as specific inhibitors of IKKβ. Figures were prepared using program PyMOL [131,132].

Evidently, the potential of IKKβ inhibition as a therapeutic strategy remains unrealized and previous optimism for IKKβ as a therapeutic target has significantly cooled. There are several plausible reasons for this lack of clinical success. The simplest is that IKKβ inhibitors developed to date may not exhibit the combination of properties required to achieve success during preclinical development, including but not limited to: nanomolar-range potency, high selectivity over IKKα/other kinases, and clinically relevant pharmacokinetics/pharmacodynamics. As mentioned earlier, several ‘selective’ IKKβ inhibitors that continue to be used by the research community have been shown to be anything but selective. IKKβ inhibitors described to date were identified following hit-to-lead development and characterization of structure–activity relationships in the absence of resolved crystal structures for the IKKs. However, the recent reports of human IKKβ X-ray crystal structures have revealed many new structural details at a sufficiently high resolution that will hopefully facilitate the structure-guided design of next-generation IKKβ inhibitors with enhanced potency and selectivity (Figure 2 and Figure 3; [5,6]).

These structures identified opportunities to rationally design highly selective non-ATP competitive inhibitors. For example, Liu et al. captured an asymmetric dimer of human IKKβ at a resolution of 2.8A, with one protomer in an active and the other in an inactive conformation, each with phosphorylated and unphosphorylated S177/S181 residues, respectively [5]. The binding mode of an inhibitor within the ATP-binding site of IKKβ was essentially identical regardless of the activation state of the kinase domain (KD), suggesting that ATP-competitive compounds are unlikely to selectively capture the inactive conformation. However, elsewhere the protein conformations were distinct, highlighting the potential for selective, non-ATP competitive inhibitors. Indeed, a recent study utilised a potential allosteric site identified at the KD-ubiquitin-like domain (ULD) interface (Figure 3A) to perform a virtual screen for allosteric inhibitors [130]. They identified a lead compound (3,4-dichloro-2-ethoxy-*N*-(2,2,6,6-tetramethylpiperidin-4-yl) benzenesulfonamide), which inhibited TNFα-induced NF-κB transcriptional activity through selective capture of the inactive conformation and hence blockade of IKKβ S177/S181 phosphorylation. The selectivity of this compound over IKKα was, unfortunately, not assessed. However, it is interesting to note that X-ray and cryo-EM structures of human IKKα demonstrated pronounced differences between IKKα and IKKβ in the orientation of the KD relative to the α-helical scaffold/dimerization domain (SDD) and its stably associated ULD, suggesting that the KD-ULD interfaces of IKKβ and IKKα may be sufficiently unique to facilitate the design of highly selective IKK inhibitors [7]. Meanwhile, Polley et al. demonstrated that the introduction of mutations within the two oligomerization interfaces identified in their constitutively active (S177E/S181E) human IKKβ X-ray crystal structure—the SDD-SDD interface of dimeric IKKβ (Figure 3B,C), and the KD-KD interface of oligomeric IKKβ (Figure 3D,E)—was sufficient to interfere with IKKβ activation/catalytic activity in vitro, indicating that small molecules designed to interfere with IKKβ oligomerization through these interfaces may function as highly selective inhibitors of IKKβ [6].

Another reason for the lack of success of IKKβ inhibitors in pre-clinical development may be their inappropriate therapeutic application. In the simplest sense this could reflect use of the wrong dosing strategy (drug concentration/dosing schedule, etc.) for effective target inhibition. More challenging is the appropriate selection of patient subgroups to achieve the desired therapeutic efficacy. For example, the proof of concept study for SAR113945 failed to show any effect in the overall group of recruited study participants for the primary endpoint; however, post-study analysis demonstrated a statistically significant difference in a patient subgroup that had presented with synovial effusion at baseline [127]. Stratification of patient subgroups to identify those with a clear NF-κB-driven, inflammatory phenotype that would benefit from IKKβ inhibition may be one solution to this problem.

A further underappreciated factor that may have impacted the clinical success of IKKβ inhibitors is the relative contribution of IKKα and/or IKKβ to the disease state being targeted. As already discussed, IKKβ has historically been considered the primary viable target to inhibit pathogenic canonical NF-κB signalling due to seminal knock-out experiments in murine cells. However, this isoform-specific delineation of function has turned out to be overly simplistic. Increasingly, it appears that in certain human cell-types IKKα may play a substantial role, alongside IKKβ, in activation of canonical NF-κB signalling, both under physiological or pathological conditions [133,134,135] and as an adaptive response to inhibition of IKKβ [136]. The relative contribution of IKKα and IKKβ to disease-associated canonical NF-κB signalling should therefore be assessed prior to any decisions to apply IKKβ-, IKKα- or dual-selective inhibitors. For example, dual siRNA-mediated knockdown of IKKα and IKKβ or dual IKKα/β inhibition had a greater suppressive effect on canonical NF-κB activation and proliferation, survival and migration of head and neck squamous cell carcinoma (HNSCC) cells than knockdown or inhibition of each IKK individually [134].

Many of these issues are likely compounded by the lack of widespread use of isoform-specific readouts of cellular IKK activity in pre-clinical studies. Non-canonical NF-κB stimuli/FBS-induced phosphorylation of human p100 at S866/870 may be used as a cellular readout of IKKα-induced non-canonical NF-κB signalling [137]. Meanwhile, the relative contributions of IKKα and IKKβ to canonical NF-κB signalling (typically assessed by measurement of phosphorylation of IκBα at S32/36 and p65 at S468 and/or S536) may be assessed using a combination of highly selective IKKβ inhibitors (such as BI605906) and siRNA or CRISPR-Cas9-mediated genetic ablation of the IKKs. IKKα-selective inhibitors may also be commercially available in the near future [35].

## 5. Safety Concerns Surrounding the Therapeutic Use of IKKβ Inhibitors

Irrespective of the reasons for the lack of clinical success of IKKβ inhibitors to date, there are several real and perceived concerns surrounding the safety of systemic administration of IKKβ inhibitors that have contributed to a significant decrease in interest in their clinical development.

One of the first observations to raise concerns about the safety of systemic IKKβ/NF-κB inhibition was the marked increase in susceptibility to apoptosis that accompanied genetic ablation of NF-κB pathway components. For example, *ikkb^-/-^* and *rela^-/-^* mice exhibit embryonic lethality due to severe liver apoptosis, which results from an absence of survival signalling in response to TNFα stimulation [28,29,138,139]. Meanwhile, enterocyte-specific ablation of IKKβ in a mouse model of gut ischemia-reperfusion resulted in severe apoptotic damage to the intestinal mucosa, highlighting a role for NF-κB signalling in maintaining tissue homeostasis in the adult mouse [140]. Curiously, however, the consequences of a lack of IKKβ in humans are strikingly different; homozygous deletion of the IKBKB gene is not embryonic lethal—at least in patients examined to date—but leads to a lack of regulatory (Treg) and γδ T cells, defects in T- and B-cell activation and to SCID associated with early infections with various pathogens [141,142,143,144]. These differences could relate to compensatory IKKα-dependent canonical NF-κB signalling in humans in certain cell types, or other species variations, and imply there may be broader differences in the response of mice and humans to systemic IKKβ inhibition. Nevertheless, these genetic studies highlight another concern with systemic IKKβ inhibition: the potential for increased susceptibility to infection due to the vital role for NF-κB signalling in the host defence system [145,146].

Additional safety concerns surrounding systemic IKKβ inhibition are largely related to the complexity of function of IKK/NF-κB signalling in inflammation and, in turn, inflammatory diseases and cancer; NF-κB signalling is often described as a ‘double-edged sword’, having pro- and anti-inflammatory functions in different contexts. Further complexity is introduced by the context-dependent roles of the NF-κB-mediated inflammatory response itself in tumorigenesis. As an example, immune-cell infiltration of tumours can have a dual role: either leading to an anti-tumour response, or immune evasion and active promotion of tumorigenesis. For a comprehensive review of this complex subject, see [147,148].

Chronic NF-κB activity has been implicated in the pathogenesis of many inflammation-related diseases [149,150] and genetic mutations that lead to increased NF-κB activity often trigger chronic inflammation and associated pathologies [151,152,153]. Indeed, NF-κB is one of the key links between chronic inflammation and cancer, as shown by the critical role of NF-κB in inflammation-driven, colitis-associated cancer (CAC) and hepatocellular carcinoma (HCC) [154,155]. Beyond maintaining a chronic inflammatory microenvironment, NF-κB has established tumour promoting roles in various cancers through the aberrant regulation of genes that influence all of the hallmarks of cancer. Many studies have confirmed that NF-κB inhibition through genetic ablation or small-molecule inhibition of IKKβ has anti-inflammatory and/or anti-tumorigenic effects in vivo [154,156,157].

However, several studies have surprisingly shown that IKKβ inhibition in certain cells/tissues triggers the spontaneous development of severe inflammatory conditions, and in some cases promotes malignant development, indicating anti-inflammatory and tumour suppressor roles for NF-κB; it appears, therefore, that the consequence of IKKβ deletion and NF-κB inhibition is highly cell-type and context dependent [158]. For example, a series of seminal studies demonstrated a role for NF-κB signalling in the negative control of inflammasome-dependent IL-1β secretion [159,160]. Hematopoietic-cell specific ablation of IKKβ or prolonged, systemic IKKβ inhibition in mice resulted in enhanced inflammasome-dependent, caspase-1-mediated IL-1β production and hyper-susceptibility to septic shock-induced endotoxin challenge or bacterial infection [159]. In turn, the enhanced IL-1β secretion following systemic IKKβ inhibition was found to promote the proliferation of granulocytic progenitors and increase the survival of mature neutrophils leading to neutrophilia and inflammatory destruction of tissues [160]. The mechanism for this NF-κB-dependent inhibition of the inflammasome remained obscure until recently when it was proposed that NF-κB promotes the expression of sequestosome-1/p62 (SQSTM1, here referred to as p62) in macrophages to facilitate p62-dependent elimination of damaged mitochondria, which act as cell-intrinsic inflammasome activating signals [161]. The clinical relevance of these observations is supported by the observation of enhanced inflammation and neutrophilia during human phase I trials of IKKβ inhibitors. Furthermore, IL-1β protein is significantly increased in the plasma of advanced non-small-cell lung carcinoma (NSCLC) patients treated with Bortezomib (trial protocol: NCT01633645) [162]. While IKKβ^Δ^ IL-1βR1^-/-^ mice do not display neutrophil-driven inflammation they exhibit severely compromised innate immunity and susceptibility to bacterial infection [160]. Furthermore, IL-1β is required for immunogenic cell death (ICD)-mediated dendritic cell (DC) maturation and antigen presentation as part of the adaptive immune response [163]. Combined inhibition of IKKβ and IL-1β signalling is, therefore, unlikely to be viable, necessitating an alternative solution to the toxicity issues of systemic IKKβ inhibition, unless coupled with intense medical supervision and strong antibiotics.

In addition, there are concerns that systemic IKKβ inhibition might promote malignant development in tissues/contexts where IKKβ/NF-κB activity plays a dominant tumour suppressor role. For example, IKKβ has been shown to act as a tumour suppressor in cancer-associated fibroblasts (CAFs) during intestinal tumorigenesis [164]. However, these findings directly contrast with other studies where IKKβ ablation in mesenchymal cells protected against inflammation-induced intestinal carcinogenesis [165,166]. The reasons for these differences are unclear but may be due to temporal differences in IKKβ inactivation and/or the targeting of different mesenchymal cell subpopulations in each model. The outcome of IKKβ ablation often depends on the targeted cell type. For example, in melanoma IKK has both tumour-promoting activity in melanocytes [156] and tumour-suppressive activity in myeloid cells [167]. Furthermore, IKKβ doesn’t always act as a ‘real’ tumour suppressor. For example, in models of chemically-induced HCC (which is not accompanied by chronic inflammation) NF-κB inactivation through liver-targeted deletion of IKKβ strongly enhances diethylnitrosamine (DEN)-induced carcinogenesis [168]. However, IKKβ inhibition only potentiates HCC under conditions of elevated hepatocyte injury, indicating that IKKβ is not a true tumour suppressor in this context. Indeed, mice with hepatocyte-specific expression of constitutively active IKKβ exhibit enhanced HCC development [169].

These examples highlight that the biological determinants influencing the pro- or anti-inflammatory/tumorigenic roles of IKKβ are complex and ill-defined. Further work is needed, both in animal models and patient tumoral samples, to unravel the context-dependencies of IKKβ functions in inflammation/cancer to better define the circumstances where the therapeutic benefits of systemic IKKβ inhibition may outweigh the risk of potential side-effects.

## 6. Recent Therapeutic Opportunities to Target IKKβ

Despite shifts in the priorities of the pharmaceutical industry away from the development of IKKβ inhibitors there are several active research areas where IKKβ remains a highly attractive clinical target. Furthermore, therapeutic strategies such as rational combination therapies and targeted delivery may be able to mitigate some of the host toxicity observed following systemic delivery of IKKβ inhibitors. Here we discuss some of these recent therapeutic opportunities to target IKKβ.

### 6.1. Cancers Exhibiting Clear ‘Addiction’ to Canonical NF-κB Signalling

While the inflammatory side-effects associated with prolonged IKKβ inhibition may limit systemic application of IKKβ inhibitors in the treatment of chronic inflammatory and autoimmune diseases, a partial and/or short-lived inhibition of IKKβ, which is unlikely to trigger widespread neutrophilia, could still find utility in the treatment of cancers in which NF-κB plays a clear initiating/driving role in tumorigenesis. The priority would be tumours bearing oncogenic, NF-κB-activating lesions, followed by tumours with constitutive NF-κB activation due to factors within the tumour microenvironment (TME). In either case, NF-κB activation must be strongly correlated with poor prognosis. In other words, identification of cancers exhibiting a wide therapeutic window of opportunity may enable partial/short-term IKKβ inhibition to have a preferential effect on malignant cells relative to normal host cells. In this context, it is important that tumour subtypes exhibiting addiction to the NF-κB pathway are identified. This can be achieved using whole genome sequencing/copy number analysis to identify relevant NF-κB component lesions alongside expression profiling of NF-κB target genes to identify gene signatures diagnostic of NF-κB pathway addiction [170,171]. A full account of the link between NF-κB and cancer is beyond the scope of this review, and readers are directed elsewhere [22,148]. However, a few illustrative examples will be discussed to highlight those cancers for which IKKβ inhibition offers the greatest therapeutic potential.

Both cell-intrinsic and -extrinsic factors contribute to aberrant NF-κB activity. Enhanced NF-κB activity can be a direct consequence of mutations of NF-κB pathway components and/or upstream oncogenes that activate NF-κB. On the other hand, a tumour can acquire elevated NF-κB activity through interaction with the inflammatory milieu of the TME. Lymphoid malignancies, particularly B-cell lymphomas, frequently exhibit direct mutations of NF-κB signalling genes [172]. For example, the pathogenesis of activated B cell-like Diffuse large B-cell lymphoma (ABC DLBCL) involves oncogenic activation of various upstream NF-κB pathway components, including the CD79B subunit of the BCR, CARD11 and MyD88, which drive cancer cell proliferation/survival through canonical NF-κB activity [171,173]. Oncogenic addiction of ABC DLBCL cells to high NF-κB activity has been demonstrated by the selective cytotoxicity of IKKβ inhibitors, providing a clear rational for therapeutic strategies targeting IKKβ/NF-κB [62,170].

In contrast, with a few exceptions [174,175,176], solid malignancies rarely exhibit direct oncogenic mutations of NF-κB pathway components. In most solid tumours NF-κB is constitutively activated due to chronic pro-inflammatory signalling within the TME. However, certain oncogenes can also drive downstream NF-κB activity. For example, studies of mouse models of Kirsten rat sarcoma viral oncogene homolog (KRAS; G12D)-induced lung cancer have demonstrated that KRAS activates NF-κB in lung tumours in situ [177,178,179]. Various mechanisms have been proposed for KRAS-mediated activation of NF-κB, including cell-autonomous feed-forward activation of PI3K-AKT, MEK-ERK and DNA-damage response signalling pathways and feed-forward autocrine signalling [157,179]. Concomitant loss of p53 activity dramatically enhances the activation of NF-κB in lung cancer cells [177,179]. Significantly, IKKβ-dependent NF-κB activation in these models has been shown to drive lung tumourigenesis, primarily through enhanced cancer cell proliferation [65,178,179]. Furthermore, KRAS-driven NF-κB activation has been associated with feedforward amplification of RAS signalling, drug resistance, and tumour stemness [180,181]. These studies collectively identify IKKβ inhibition as a promising therapeutic strategy in KRAS-driven lung cancer with altered p53 activity [65]. It should be noted, however, that several studies have also linked KRAS-driven IKKα and TBK1 activation to pathogenic NF-κB activity [181,182,183,184], suggesting that IKKβ inhibition alone may not be an optimal therapeutic strategy.

A potentially crucial aspect of IKKβ inhibition as a cancer therapy is the appropriate timing of treatment with respect to the stage of cancer progression. Given the potential toxicity of IKKβ inhibition towards T cells, such treatment is likely to be undesirable during the early tumour-eliminating phase of the immune system, when cytotoxic T lymphocytes (CTLs) target transformed cells. Rather, based on current knowledge, IKKβ inhibition is more likely to have positive therapeutic effects in the chronic inflammatory phase of tumour progression [22]. However, this generalisation may be an oversimplification (see Section 6.3) and may not apply to all cancers. Indeed, given the context-dependency of the pro- and anti-inflammatory effects of IKKβ inhibition, it remains to be determined what the net effect systemic, pharmacological IKKβ inhibition may have in different cancers.

### 6.2. Use of IKKβ Inhibitors in Combination Therapies to Combat Chemoresistance

The consensus from pre-clinical studies is that IKKβ inhibitors are unlikely to achieve broad clinical success as single agents in cancer therapy, except perhaps for certain types of lymphoma/leukaemia. However, there is significant optimism that they might yet enter the clinic as part of combination therapies with conventional therapeutics (chemo- and radiotherapeutics), and certain targeted therapies, in cancers where NF-κB signalling has been associated with chemoresistance [185]. Use of synergistic combinations may enable lower concentrations of IKKβ inhibitors to be employed to achieve a desired therapeutic effect, thus reducing systemic toxicity.

The cytotoxic/cytostatic effects of conventional therapeutics, such as cisplatin and radiotherapy, typically rely on their ability to preferentially induce DNA damage in highly proliferative cancer cells, which triggers cell cycle arrest and, ultimately, cell death or senescence. NF-κB activity is associated with chemoresistance in various cancers through the induction of genes involved in the control of survival, proliferation, inflammation, DNA repair, metabolic reprogramming, angiogenesis, drug uptake/inactivation, etc., that reduce the efficacy of conventional therapeutics [186]. Intrinsic chemoresistance has been correlated with constitutive NF-κB activation in numerous cancers [187,188,189]. Chemoresistance is also commonly acquired or enhanced by therapy-induced NF-κB activation [186,190,191]. For example, genotoxic agents that induce double-strand DNA breaks (DSBs) activate IKK-dependent NF-κB activity via a NEMO-ATM dependent pathway initiated in the nucleus (Figure 1E; [192]). As such, IKKβ inhibitors are under investigation as a route to sensitize cancer cells to conventional genotoxic therapeutics. For example, inhibition of IKKβ with MLN-120B leads to synergistic enhancement of vincristine cytotoxicity in non-Hodgkin’s lymphoma via suppression of vincristine-induced NF-κB activity [193]. Interest in IKK/NF-κB inhibition is likely to enhance as ever greater numbers of studies/clinical trials identify therapy-induced NF-κB activity as a key factor in the relapse response to conventional therapies (Identifier: NCT00280761).

IKKβ inhibitors are also being investigated in combination with certain targeted therapeutics to overcome intrinsic resistance. For example, activation of NF-κB has been strongly linked to intrinsic and acquired resistance to epidermal growth factor receptor (EGFR) inhibitors [181,194,195], while synergistic cytotoxicity has been observed in ABC DLBCL treated with JAK and IKKβ inhibitors [196]. However, it should be noted that, in general, the IKK isoform-dependence of NF-κB-mediated chemoresistance mechanisms has not been characterised. IKKβ inhibition may not always be the most effective means to counteract chemoresistance. Indeed, dual IKKα/β inhibition may be more effective than IKKβ inhibition alone in the counteraction of resistance to EGFR inhibitors in HNSCC [134].

### 6.3. IKKβ Inhibitors as an Adjunct to Cancer Immunotherapies

Recent reports suggest IKKβ inhibitors may also combine well with certain cancer immunotherapies. Cancer immunotherapy is a general term for treatments that harness or reactivate the body’s own immune system to target and destroy tumours. For instance, this may be a tumour-specific vaccination that enhances the anti-tumour activity of the patients own CTLs. However, CTL-dependent anti-tumour responses are often suppressed by FOXP3+ Tregs that infiltrate tumours. Therefore, several cancer immunotherapies that inhibit or deplete FOXP3+ Treg cells are currently being tested [197]. As described earlier, patients with rare homozygous deletion of the IKBKB gene typically lack Treg cells [141]. Consistent with this, prolonged IKKβ inhibition was recently shown to partially deplete circulating FOXP3+ Treg cells, due to their dependence on NF-κB signalling for survival [198]. Consequently, administration of an IKKβ inhibitor after tumour vaccination in a mouse melanoma model enhanced the CTL-dependent anti-tumour response and delayed tumour growth, identifying IKKβ as a potential druggable immune checkpoint. A large caveat, however, is that the correct dosage or scheduling of IKKβ inhibition is likely to be critical to achieve potentiation, rather than inhibition of the anti-tumour response, due to the importance of NF-κB activity for T cell survival/function [31]. For example, genetic deletion of IKKβ in T-cells abrogated the anti-tumour response in mice with fibrosarcoma [199]. Indeed, Heuser et al. observed that high doses of IKKβ inhibitor suppressed CTL responses in their model [198]. This dosage effect was explained by the fact that Tregs were more sensitive than CTLs to IKKβ inhibition due to a greater reliance on NF-κB signalling for survival. These findings also suggest further caution is warranted in attempts to utilise systemic IKKβ inhibition to treat inflammatory diseases.

A key feature of tumour immune evasion is the increased expression of certain ligands, notably programmed cell death-ligand 1 (PD-L1), at the cell surface of cancer cells and immune cells, such as dendritic cells and macrophages [200,201]. Binding of PD-L1 to its cognate receptor, PD-1, on T cells inhibits their proliferation/survival and effector cytokine secretion to downregulate CTL responses [202,203]. As such, anti-PD-1/PD-L1 therapies have now demonstrated marked success in a wide range of malignancies [204]. However, high expression of surface PD-L1 by solid tumours and tumour-infiltrating myeloid cells appears to be correlated with poor prognosis in certain cancers and may negatively impact the clinical response to PD-1 blockade [205]. The mechanisms regulating PD-L1 expression are, therefore, of clinical interest. Interestingly, NF-κB signalling has been shown to promote the expression of PD-L1 at the transcriptional [206,207] and, more recently, the post-transcriptional level [208]. Lim et al. demonstrated that in an inflammation-enhanced tumour model, macrophage-derived inflammatory cytokines (such as TNFα) enhanced PD-L1 expression in cancer cells through protein stabilization via NF-κB-dependent upregulation of COP9 signalosome 5 (CSN5). Consequently, administration of curcumin (which can inhibit NF-κB and CSN5 activity directly) inhibited CSN5-dependent PDL1 stabilisation and enhanced the efficacy of anti-CTLA4 therapy. Any study that employs curcumin comes with several caveats because of the sheer number of signalling pathways this natural product deregulates, including ERK, JNK, p38 and Akt, as well as NF-κB, in addition to effects on cathepsins. Nonetheless, this approach has received some support by the observation that TNFα-blockade overcomes resistance to anti-PD-1 in experimental melanoma [209]. Whether these strategies might also apply to IKKβ-selective inhibitors warrants further investigation.

Another form of cancer immunotherapy involves the use of oncolytic viruses (OVs), which specifically infect and kill tumour cells. For example, Talimogene laherparepvec (or T-Vec), based on herpes simplex virus 1 (HSV-1), has been clinically approved for the treatment of advanced melanoma [210]. However, there is considerable heterogeneity in the therapeutic response to OV therapy, with resistance largely due to failure of tumour cells to become infected by the virus [211]. Interestingly, dimethyl fumarate (DMF) was recently shown to enhance OV infection of cancer cells and improve therapeutic outcomes in resistant syngeneic and xenograft tumour mouse models through inhibition of NF-κB signalling and, in turn, the antiviral response of cancer cells [212]. IKKβ-selective inhibitors also improved OV infection in vitro [50,212,213], suggesting a combination of oncolytic virotherapy and IKKβ inhibitors may warrant further clinical investigation.

Finally, there are reports that IKKβ inhibition may enhance anti-tumour immunity through modulation of the activity of tumour-associated macrophages (TAM). A simplified model states that TAMs recruited to tumours characterised by non-resolving inflammation switch from a tumour-killing, ‘classically activated’ M1-like phenotype to a tumour-promoting, ‘alternatively activated’ M2 phenotype that stimulates cancer cell proliferation, angiogenesis and metastasis and suppresses immune effector cells [214]. The modulation of TAM survival/polarization is, therefore, an attractive therapeutic target [215]. Inhibition of NF-κB activation in TAMs through macrophage-specific ablation of IKKβ has been proposed to promote polarization to the anti-tumour M1 phenotype, thereby enhancing tumour regression [216]. However, there are other conflicting reports that suggest that M2 immunosuppressive TAMs from established tumours are characterised by defective NF-κB transcriptional responses, due, in part, to overexpression of repressive p50 homodimers [217,218]. In turn, enforced re-activation of NF-κB can polarize M2-like TAMs to an M1-like phenotype and induce their tumour cytotoxicity [219,220,221]. Various explanations have been proposed for these contrasting findings [222]. As discussed earlier in the broader context of cancer therapy, the clinical outcome of pharmacologic targeting of macrophage IKKβ/NF-κB activities may depend on the disease stage; that is, premalignant, malignant or metastatic. This example is also representative of a wider issue: given the complexity of NF-κB function in the immune system it is perhaps prohibitively difficult to predict the outcome of systemic IKKβ inhibition on anti-tumour immunity, thus limiting the potential of IKKβ inhibitors as adjuncts to cancer immunotherapies.

### 6.4. Targeted Delivery of IKKβ Inhibitors to Specific Tissues

If the safety issues associated with systemic IKKβ inhibition prove insurmountable, a less toxic therapeutic approach may be the topical/local or targeted administration of inhibitors to specific disease areas. The former strategy has been demonstrated in pre-clinical studies investigating the use of IKKβ inhibitors to treat choroid neovascularization (CNV), which is a major pathological change associated with exudative age-related macular degeneration (AMD) [223]. IKKβ-dependent NF-κB signalling plays a significant role in CNV development [53]. Consequently, retrobulbar administration of IKKβ inhibitor (TPCA-1)-loaded poly-lactide-co-glycolide (PLGA) microparticles achieved controlled, durable intraocular release of drug, leading to attenuation of CNV and macrophage recruitment, without systemic toxicity in a laser-induced mouse model of CNV [223].

Furthermore, as discussed in Section 5, numerous studies have demonstrated that the therapeutic outcome of IKK/NF-κB inhibition in a specific cancer model depends on the cell-type(s) selected for genetic ablation of the NF-κB signalling component. For instance, in a mouse melanoma model, ablation of tumour-intrinsic NF-κB activity using cancer cell-targeted expression of an IκBα super-repressor construct led to cytotoxicity-driven tumour regression following doxorubicin treatment [224]. However, myeloid-specific loss of NF-κB signalling resulted in increased host toxicity and mortality without tumour regression due to excessive IL-1β production, consistent with other studies [159]. Results such as this strongly advocate for a targeted delivery approach to IKKβ inhibition to reduce therapy-associated systemic toxicity. αvβ3-ligand tetraiodothyroacetic acid (TET)-modified micelles have been shown to promote selective accumulation of the NF-κB inhibitor, celestrol, in primary tumours and lung metastases and to inhibit tumour growth and metastasis in a breast cancer mouse model to a greater extent than systemic delivery of celestrol alone [225]. Although relative safety profiles were not assessed in this case, this study demonstrates a promising proof-of-principle.

## 7. Alternative Approaches to Targeting the NF-κB Pathway

As the ubiquitously expressed, primary, druggable mediator of canonical NF-κB signalling, IKKβ was the logical, first-choice target for the development of pharmacological inhibitors of the NF-κB pathway. As we have discussed, IKKβ inhibitors have displayed significant therapeutic potential, and new developments continue to identify IKKβ as an attractive therapeutic target. However, the potential risks of on-target systemic toxicity, immunodeficiency and malignant development arising in contexts where IKKβ/NF-κB activity plays a dominant tumour suppressor role may prove insurmountable and forever undermine research efforts to clinically develop IKKβ-targeting therapeutics. Some, if not all, of these safety concerns may be circumvented by targeting alternative nodes in the NF-κB pathway [226,227]. Indeed, this strategy has seen far greater clinical success than directly targeting IKKβ. A few illustrative examples will be discussed here.

### 7.1. Targeting Upstream NF-κB Signalling Components

Multiple signalling pathways converge to activate NF-κB. Inhibition of disease-specific upstream NF-κB components may provide a greater degree of tissue- and context-specificity than directly targeting IKKβ, which is activated by all NF-κB-inducing signals. A clinically-relevant example is the specific targeting of BCR-induced NF-κB signalling (see Figure 1D) in B-cells using inhibitors of Burton tyrosine kinase (BTK), such as Ibrutinib, which is approved for the treatment of refractory mantle cell lymphoma (MCL) [228] and marginal zone lymphoma (MZL) [229], chronic lymphomatic leukemia (CLL) [230], small lymphocytic lymphoma (SLL) [230], Waldenström’s macroglobulinemia [231] and chronic graft versus host disease [232] The highly-restricted pattern of BTK expression to B cells means that Ibrutinib has a generally well-tolerated safety profile.

### 7.2. Targeting IKKα or NEMO

Until recently IKKα has received minimal attention as a target for drug development [35]. However, IKKα is an essential component of the non-canonical NF-κB pathway, making it an attractive therapeutic target in diseases where aberrant activity of this pathway contributes to pathogenesis, such as mucosa-associated lymphoid tissue (MALT) lymphoma and MM [233]. Furthermore, the growing realisation that IKKα, alongside IKKβ, contributes to canonical NF-κB pathway regulation in certain contexts should motivate the investigation of IKKα-selective or IKKα/β dual selectivity inhibitors in diseases where canonical NF-κB signalling plays a role in pathogenesis [134]. Equally, selective targeting of IKKα may enable context-specific inhibition of pathogenic NF-κB signalling, while retaining sufficient ‘physiological’ levels of canonical NF-κB signalling in host tissues to prevent the adverse effects seen with IKKβ inhibitors. IKKα also has numerous NF-κB-independent functions that could be targeted (see Section 7.3).

Significantly more effort has been devoted to development of molecules that target the NEMO scaffold, which is essential for canonical NF-κB signalling [234]. IKKα and IKKβ interact with NEMO via their short, C-terminal NBDs, consisting of the hexapeptide amino acid sequence, LDWSWL. Peptides corresponding to the NBD domain fused to sequences that facilitate intracellular delivery disrupt the association of IKKs with NEMO and block NF-κB transcriptional activation in cells [107]. NBD peptides have exhibited promising therapeutic effects in numerous disease models [235]. For example, a recent phase I trial of systemic administration of NBD peptides for the treatment of dogs with spontaneous ABC DLBCL demonstrated safety and treatment efficacy, offering hope for translation of this therapy to human ABC DLBCL [236]. The safety/toxicity profile of NBD peptides are generally more favourable than IKKβ inhibitors, potentially because they suppress only stimulus-induced rather than basal, IKK activity [107]. They are also less likely to inhibit ‘off-target’ kinases due to the specificity of the protein interaction between NBD peptides and NEMO. Clinical success may be limited, however, by the expense of peptide synthesis, their short half-life, and poor oral bioavailability, requiring intravenous administration. Small-molecule NBD mimetics that could overcome these limitations have recently been described [225].

Recently, NEMO-ubiquitin interaction inhibitors (iNUBs) have been characterised [237]. iNUBs inhibited IKK/NF-κB activation and target gene expression in response to TNFα, but not IL-1β, stimulation. iNUBs, therefore, may offer a strategy to selectively impair NF-κB signalling in response to specific stimuli.

### 7.3. Targeting NF-κB-Independent Functions of the IKKs

As described in Section 1, the IKKs exert multiple NF-κB-independent functions through the phosphorylation of so-called ‘non-classical’ substrates. Many of these activities have been proposed to contribute to disease progression [238]. Targeting these NF-κB-independent functions may, therefore, offer an opportunity to block IKK-driven pathogenesis without the side-effects associated with systemic NF-κB inhibition. For example, IKKα has been proposed to promote mammalian target of rapamycin complex 1 (mTORC1) and mTORC2 activity via feedforward signalling, and thus could serve as a therapeutic target in mTOR-dependent cancers in a manner independent of potent canonical NF-κB inhibition [239,240]. Meanwhile, IKKβ phosphorylates FOXO3a at Ser644 to promote its nuclear exclusion and ubiquitination-mediated proteasomal degradation to promote breast cancer progression [26]. However, further work is needed to validate many of these targets as bona fide IKK substrates and to understand the importance of these NF-κB-independent functions of the IKKs in disease progression. Characterisation of NF-κB-independent functions is also vital in order to anticipate and explain the potential side effects of targeting IKK kinase activity.

### 7.4. Targeting IκBα Degradation

The ubiquitination and proteasomal degradation of IκB proteins represents an essential step in the activation of NF-κB pathways. Inhibitors of the ubiquitin-proteasome system (UPS), therefore, inhibit NF-κB activity by stabilising IκB proteins. A prominent example is the first-generation proteasome inhibitor, bortezomib, which has achieved significant clinical successes, particularly in relapsed and/or refractory MM and MCL [241,242]. However, despite these successes, therapeutic proteasome inhibitors have several limitations, including dose-limiting toxicity, the rapid onset of secondary drug resistance and broad cellular activities, which often lead to unpredictable outcomes. It is also unclear to what extent the clinical response to proteasome inhibitors in lymphomas results from the inhibition of NF-κB signalling [243]. Proteasome inhibitors will impinge on a legion of critical cell regulatory pathways, so the notion that they can be used as a strategy to selectively target NF-κB pathways is, at best, fanciful. Indeed, malignant plasma cells are thought to be more sensitive to proteasome inhibition than non-malignant cells because their increased rate of immunoglobulin protein production leads to enhanced cellular accumulation of undegraded, polyubiquitinated proteins in the presence of proteasome inhibitors, which activates the unfolded protein response (UPR), thereby accelerating tumour-cell apoptosis [244].

### 7.5. Targeting NF-κB Activity Directly

The activity of the NF-κB transcription factors themselves could theoretically be inhibited by several different mechanisms including blockade of nuclear translocation, dimerization and DNA binding, and inhibition of protein interactions with other essential cofactors. The reality is that drug development success in this area has been limited, mostly due to the generalised difficulty of targeting nuclear transport and of developing inhibitors of protein-protein interactions. Peptidomimetics, such as SN-50, which blocks nuclear import of p50-containing NF-κB dimers, have been widely used as research tools, but are currently unsuitable for clinical development due to their vast non-specific effects [245]. However, a collection of recent studies has reinvigorated interest in directly targeting NF-κB subunits. Conditional deletion of p65 and c-Rel in developing and mature Treg cells demonstrated their unique but partially overlapping roles in Treg cell development [246]. Subsequently, targeting c-Rel with an FDA-approved xanthine derivative, pentoxifylline (PTXF; [247]), improved checkpoint-targeting immunotherapy protocols (e.g., anti-PD1 therapy) to enhance anti-tumour immunity in a melanoma model [248]. In contrast to IKKβ inhibition [198], minimal adverse effects were observed. Because the critical biological function of c-Rel appears to be mainly restricted to the adaptive immune system, it may represent a more suitable target than IKKβ/p65 to enhance checkpoint-targeting immunotherapies [249]. In addition, IKKβ deletion in mouse bone-marrow derived hematopoietic cells results in significant defects in haematopoiesis not seen upon p65 deletion, suggesting NF-κB-independent pathways may mediate some of the hematopoietic defects associated with IKKβ deletion/inhibition [250]. This provides further rationale for targeting NF-κB subunits directly.

### 7.6. Targeting Downstream Effectors of NF-κB-Dependent Pathogenesis

It is the aberrant NF-κB-dependent regulation of specific gene expression profiles that primarily contributes to disease progression. These transcriptional programmes are often highly stimulus and tissue specific. Therefore, an attractive alternative to IKKβ inhibition is the targeting of non-redundant, disease-specific downstream mediators of pathogenic NF-κB activity. In theory, this approach should identify safer, context-specific therapeutics that preserve the multiple critical physiological functions of NF-κB. A few studies have demonstrated the exciting potential of this strategy. For example, TIMP-1 is a key downstream modulator of NF-κB-dependent tumour growth in mouse KRAS-driven lung cancer models and represents a potentially safer therapeutic target than IKKβ [179]. Another approach is to target subsets of NF-κB-controlled genes based on their dependence on specific regulatory mechanisms that are not involved in the activation of other genes. For example, the selective blockade of pro-inflammatory NF-κB-dependent transcriptional responses using inhibitors of the epigenetic modifiers bromodomain and extra-terminal (BET) proteins, such as JQ1, exhibited synergistic effects alongside JAK/STAT inhibition in myeloproliferative neoplasms [251,252].

## 8. Conclusions

As our appreciation of the fundamental role of dysregulated NF-κB signalling in the pathogenesis of inflammatory disease and cancer continues to grow so the resolve of the pharmaceutical industry to pharmacologically target NF-κB pathway components strengthens. Only a small cross-section of recent developments in the therapeutic application of IKKβ inhibitors have been discussed in this review. The number of conditions where IKKβ inhibitors are being pursued as a treatment option was too great to cover here but includes: obesity-associated metabolic disease [253], atherosclerosis [254], multiple sclerosis [255], COPD [256], muscular dystrophy [257], Parkinson’s disease [258], inflammatory bowel disease [259] and chronic arthritis [260]. However, as has often been the case in studies of the role of IKKβ in cancer progression, there have been conflicting reports of the importance of IKKβ activity in the progression of other inflammatory disorders. In the context of atherosclerosis, for example, aberrant vascular endothelial cell-specific canonical NF-κB pathway activation has been shown, quite convincingly, to promote monocyte recruitment and atherosclerotic plaque formation [261,262]. Indeed, endothelial cell-specific NEMO ablation or expression of dominant-negative IκBα protects mice from atherosclerosis [263]. The role of myeloid-specific IKKβ activity in atherosclerosis is less clear, however, as myeloid-specific IKKβ deletion has been shown to both attenuate [264] and enhance [265] atherosclerosis severity in LDL receptor-deficient mice in separate studies. These discrepancies again raise questions about whether a favourable clinical outcome could be achieved following systemic IKKβ inhibition in this disease context.

Despite promising pre-clinical results for IKKβ inhibitors in the aforementioned disease settings, there is little optimism that IKKβ inhibitors will soon broadly enter the clinic. IKKβ is ubiquitously expressed and carries out many critical physiological roles. Furthermore, the complex context- and tissue-specific functions of IKK/NF-κB signalling have made it difficult to predict the net effect and outcome of systemic intervention, and safety concerns currently appear too great a barrier to overcome. As discussed, the most compelling rationale for clinical use of IKKβ inhibitors appears to be in combination therapies and/or as part of a targeted delivery approach, particularly in cancers exhibiting clear addiction to constitutive NF-κB signalling.

If the safety concerns associated with IKKβ inhibitors are to be overcome and they are to offer lasting health benefits as a cancer therapy, research into intrinsic and acquired resistance mechanisms will also be necessary. Resistant tumours eventually develop in a mouse model of KRAS-driven, p53 mutant NSCLC treated with IKKβ inhibitors after a prolonged period of tumour-free survival [266], but no mechanisms of resistance to IKK/NF-κB inhibitors have been defined to-date. Furthermore, further work is needed to develop IKKβ inhibitors with the combination of properties necessary for clinical success, including high selectivity, nanomolar potency, a transient/reversible mode-of-action (since long-term inhibition is unfavourable), desirable pharmacokinetics/pharmacodynamics and amenability to targeted delivery.

Intensive research within the NF-κB signalling field has uncovered the complexity governing cell-type and stimulus-specific NF-κB transcriptional programs, including IKK-isoform and NF-κB-subunit specific functions. This complexity acts both as a barrier and an opportunity to develop highly selective therapies that overcome the issues inherent with systemic targeting of IKKβ.

## Figures and Tables

**Table 1:** Commercially available IKKβ inhibitors.

Inhibitor	Mechanism	Ki/IC_50_ for IKKβ (nM) * [Ref]	Selectivity Over IKKα	Known Off-Targets	Bio-Availability	Pre-Clinical Therapeutic Efficacy
BI605906 (BIX02514)	ATP-competitive	380 [40]	>300 fold (>100 µM)	>300-fold selectivity over 100 representative tyr/ser-thr kinases IGF1 (7.6 µM)	N/A	N/A
MLN120B	ATP-competitive	60 [41]	>1000 fold (>100 µM)	>1000-fold selectivity over 30 representative tyr/ser-thr kinases	Good oral bio-availability	Multiple myeloma [42] Arthritis [43]
PHA-408	ATP-competitive	10–40 [44,45]	>350 fold (14 µM)	>100-fold selectivity over 30 representative tyr/ser-thr kinases PIM-1 (0.6 µM)	Good oral bio-availability	Arthritis [44] COPD [46,47]
TPCA-1 (IKK inhibitor IV)	ATP-competitive	18 [48]	~22-fold (400 nM)	STAT3	Poor oral bio-availabilityAdministered intra-peritoneally	Arthritis [48] Nasal epithelium inflammation [49] Glioma [50] NSCLC [51] COPD [52] Wet AMD [53]
SC-514	ATP-competitive	3000–12,000 [54]	>15-fold (>200 µM)	CDK2/CycA (61 µM) Aurora A (71 µM) PRAK (75 µM) MSK (123 µM)	Poor oral bio-availabilityAdministered intra-peritoneally	Rat model of inflammation [54] Oral squamous cell carcinoma [55] Osteoclast-related disorders [56] Diabetic neuropathy [57]
LY2409881	ATP-competitive	30 [58]	> 10-fold	>10-fold selectivity over panel of representative tyr/ser-thr kinases	Administered intra-peritoneally	DLBCL [58]
PS-1145	ATP-competitive	100 [59,60]	N/A	[61]	Administered intra-peritoneally	Multiple myeloma [61] DLBCL [62] Graft-versus-host disease [60] Tobacco smoke-induced pulmonary inflammation [63]
Compound A (Bay 65-1942)	ATP-competitive	Ki for GST-IκBα = 4 nM [64]	>30 fold (135 nM)	IKKε, MKK4, MKK7, ERK-1, Syk, Lck, Fyn, PI3Kγ, PKA and PKC (IC50 > 10 µM)	Good oral bio-availability	KRAS-induced lung cancer [65] Chronic pulmonary inflammation [64] Ischemia–reperfusion injury [66] LPS-induced neurotoxicity [67]
IKK-16 (IKK Inhibitor VII)	ATP-competitive	40–70 [68,69]	5-fold (200 nM)	LRKK2 (50 nM)	Good oral bio-availability	Multiple organ failure associated with hemorrhagic shock [70] Sepsis-associated multiple organ dysfunction [71] Ventilation-induced lung injury [72] Acute kidney injury [73]
IMD-0354 (and pro-drug IMD-1041)	ATP-competitive	~1µM [74,75]	N/A	N/A	Administered intra-peritoneally	CLL [76] Pancreatic cancer [77] Adult T-cell leukemia [78] Breast cancer [75]
ACHP (IKK inhibitor VIII)	ATP-competitive	8.5 [79]	30-fold (250 nM)	IKKε, Syk, MKK4 (IC_50_ > 20 µM)	Good oral bio-availability	Multiple myeloma [80] Adult T-cell leukemia [81] HIV-1 replication [82]
BMS-345541	Allosteric	300 [83]	~13-fold (4000 nM)	>300-fold selectivity over a small panel of representative tyr/ser-thr kinases	Good oral bio-availability	Arthritis [84] Colitis [85] Cardiac graft rejection [86] T-ALL [87] Glioma [50] Prostate cancer [88]
Withaferin A	Cys179-binding	[89,90,91,92]	N/A	Broad spectrum inhibitor [93]Vimentin, HSP90, β-tubulin, Desmin, Annexin-A2, Notch-1, STAT1/3	Poor oral bioavailability	N/A
BOT-64	Ser-177/181 binding	1000–3000 [94]	N/A	N/A	Administered intra-peritoneally	N/A
Ainsliadimer A	Cysteine-46 binding	30 [95]	N/A	No significant activity against 340 human kinases at 200 nM	Administered intravenously	N/A

* Value as reported in the reference, from activity or binding assay, not corrected for ATP concentration.

## Abbreviations

The following abbreviations are used in this manuscript

ABC DLBCL	Activated B cell-like Diffuse large B-cell lymphoma
BCR	B-cell receptor
BET	Bromodomain and extra-terminal
BTK	Burton tyrosine kinase
β-TrCP	Beta-transducing repeat-containing protein
CAC	Colitis-associated carcinoma
CAF	Cancer-associated fibroblast
CLL	Chronic lymphomatic leukemia
CNV	Choroid neovascularization
COPD	Chronic obstructive pulmonary disease
CSN5	COP9 signalosome 5
CTL	Cytotoxic T-lymphocyte
DC	Dendritic cell
DEN	Diethylnitrosoamine
DMF	Dimethyl fumarate
DSB	Double-strand break
EGFR	Epidermal growth factor receptor
HCC	Hepatocellular carcinoma
HNSCC	Head and neck squamous cell carcinoma
ICD	Immunogenic cell death
IKK	IκB kinase
IL-1	Interleukin-1
iNUB	Inhibitor of NEMO-Ubiquitin binding
IκB	Inhibitor of kappa B
KD	Kinase domain
KRAS	Kirsten rat sarcoma viral oncogene homolog
LPS	Lipopolysaccharide
LUBAC	Linear ubiquitin chain assembly complex
MALT	Mucosa-associated lymphoid tissue
MCL	Mantle cell lymphoma
MM	Multiple myeloma
mTORC	Mammalian target of rapamycin complex
NBD	NEMO-binding domain
NF-κB	nuclear factor- “kappa-light-chain-enhancer” of activated B-cells
NSCLC	Non-small-cell lung carcinoma
OV	Oncolytic virus
PD/PDL	Programmed death/PD-ligand 1
POC	Proof-of-concept
RA	Rheumatoid arthritis
RHD	Rel homology domain
SCF	S phase kinase-associated protein 1 (SKP1)-cullin 1-F-box protein
SCID	Severe combined immunodeficient
SDD	Scaffold/dimerization domain
SH2	Src Homology 2
TAM	Tumour-associated macrophage
TCR	T-cell receptor
TLR	Toll-like receptor
TME	Tumour microenvironment
TNFα	Tumor necrosis factor-alpha
Treg	Regulatory T cells
UBC	Ubiquitin-conjugating enzyme
ULD	Ubiquitin-like domain
